# On the Treatment of Pneumonia by the Inhalation of Chloroform

**Published:** 1852-07

**Authors:** 


From Henle & Pfeufer's Zeitschrift, N. S. band i. p. 76.

*On the Treatment of Pneumonia by the inhalation of Chloroform.* By Dr. Varrentrapp.

Although Dr. Varrentrapp is well aware that the 23 cases of which he furnishes the history in his paper, are too few in number to justify him in drawing any positive conclusions from them, he thinks that the results are sufficiently striking to justify his adding them to those already published by Wucherer and Baumgertner, which indeed he considers have not excited attention in proportion to their importance. His own cases differed from theirs, in his employing larger and more frequent doses of, and trusting exclusively to, the chloroform. The cases were composed of the working classes who frequent the Frankfort hospital, the average age of the 23 persons (21 men and 2 women) being 31, the eldest 62, and the youngest 19. Upon the average, the treatment was commenced on the morning of the fifth day of the disease. In 10 the right, in 8 the left, and in 5 both lungs were affected. The inhalations of the whole number averaged 74 for 10½ days; the smallest number being 27 in 5 days; the largest 162 in 15 days. Sixty drops of the chloroform were poured on tightly-compressed cotton, and inhaled for 10 or 15 minutes, from 8 to 12 times during the 24 hours. It seldom happened that the chloroform could not be borne on account of immediate narcotic or unpleasant symptoms; and any affections of the sensorium, vomiting, &c., that appeared at first were soon relieved by temporary suspension. Of the 23 cases, 1 was bled, and another cupped in mistake. An emetic was given in 1 case, and a purgative in 9. In 5 cases of pleuritic complication, blisters were applied, and in 2 calomel and digitalis were administered. Among the 23, 1 case was fatal, and 22 completely recovered.

